# Massively Parallel Sequencing of Genes Implicated in Heritable Cardiac Disorders: A Strategy for a Small Diagnostic Laboratory

**DOI:** 10.3390/medsci5040022

**Published:** 2017-10-10

**Authors:** Ivone U. S. Leong, Alexander Stuckey, Daniele Belluoccio, Vicky Fan, Jonathan R. Skinner, Debra O. Prosser, Donald. R. Love

**Affiliations:** 1 Diagnostic Genetics, LabPLUS, Auckland City Hospital, PO Box 110031, Auckland 1148, New Zealand; ivone.leong@ki.se (I.U.S.L.); DProsser@adhb.govt.nz (D.O.P.); 2 Bioinformatics Institute, The University of Auckland, Private Bag 92019, Auckland 1142, New Zealand; alexander.stuckey@scilifelab.se (A.S.); v.fan@auckland.ac.nz (V.F.); 3 Agilent Technologies, Mulgrave, VIC 3170, Australia; daniele_belluoccio@agilent.com; 4 Greenlane Paediatric and Congenital Cardiac Service, Starship Children’s Hospital, Grafton, Private Bag 92024, Auckland 1142, New Zealand; JSkinner@adhb.govt.nz; 5 Cardiac Inherited Disease Group, Auckland City Hospital, Auckland 1148, New Zealand; 6 Department of Paediatrics, Child and Youth Health, The University of Auckland, Private Bag 92019, Auckland 1142, New Zealand

**Keywords:** sudden cardiac death, massively parallel sequencing, next generation sequencing, cardiac gene panel

## Abstract

Sudden cardiac death (SCD) in people before the age of 35 years is a devastating event for any family. The causes of SCD in the young can be broadly divided into two groups: heritable cardiac disorders that affect the heart structure (cardiomyopathies) and primary electrical disorders (cardiac ion channelopathies). Genetic testing is vital as those suffering from cardiac ion channelopathies have structurally normal hearts, and those with cardiomyopathies may only show subtle abnormalities in the heart and these signs may not be detected during an autopsy. Post-mortem genetic testing of SCD victims is important to identify the underlying genetic cause. This is important as family cascade screening may be undertaken to identify those who may be at risk and provide vital information about risk stratification and clinical management. The development of massively parallel sequencing (MPS) has made it possible for the simultaneous screening of multiple patients for hundreds of genes. In light of this, we opted to develop an MPS approach for SCD analysis that would allow us to screen for mutations in genes implicated in cardiomyopathies and cardiac ion channelopathies. The rationale behind this panel was to limit it to genes carrying the greatest mutation load. If no likely pathogenic gene variant were found then testing could cascade to whole exome/genome sequencing as a gene-discovery exercise. The overarching aim was to design and validate a custom-cardiac panel that satisfies the diagnostic requirements of LabPLUS (Auckland City Hospital, Auckland, NZ) and the guidelines provided by the Royal College of Pathologists of Australasia and the Association for Clinical Genetic Science.

## 1. Introduction

Sudden cardiac death (SCD) in people before the age of 35 years is a devastating event for any family, and it has a yearly incidence of 0.005–0.2 per 1000 [1,2]. The heritable causes of SCD in the young can be broadly divided into two groups: cardiac disorders that affect the heart structure (cardiomyopathies), and primary electrical disorders (cardiac ion channelopathies). Cardiac ion channelopathies are commonly implicated in those under the age of 30 years; in particular, Long QT syndrome (LQTS), catecholaminergic polymorphic ventricular tachycardia (CPVT), Brugada syndrome (BrS) and, more rarely, short QT syndrome (SQTS) [2,3,4,5]. Genetic testing, combined with cardiac screening of relatives [6,7], are vital for these disorders as the heart is structurally normal and would not be diagnosed at autopsy [8,9]. Cardiomyopathies, such as hypertrophic cardiomyopathy (HCM), arrhythmogenic right ventricular cardiomyopathy (ARVC) and dilated cardiomyopathy (DCM), become more common as a cause of death from teenage years onwards [3]. These disorders exhibit morphological and histological features; however, these signs may be subtle or even absent in those that are very young. Post-mortem genetic testing (molecular autopsy) uses DNA extracted from blood/tissue of SCD victims to identify the underlying genetic cause of death in SCD cases. This is important as family cascade screening may be undertaken to identify those who may be at risk and provide vital information about risk stratification and clinical management [10].

The development of massively parallel sequencing (MPS) has made it possible to screen for hundreds of genes, whole exomes or whole genomes per patient simultaneously. There are several diagnostic laboratories already offering large gene panels for cardiac/SCD genes [11,12]; however, the likelihood of detecting mutations in some genes that feature in these lists may satisfy comprehensiveness but at a cost in terms of sequence load and variant classification. We sought to revert to empirical data and develop a gene panel that would allow us to screen for mutations in genes that, together, would give a reasonable likelihood of carrying a sequence-detectable mutation event. We chose to target those genes with the highest frequency of mutations for the following cardiac disorders: ARVC, BrS, CPVT, DCM, HCM, LQTS and SQTS (Table 1). The 29 cardiac genes included in the custom-cardiac panel would cover the diagnosis of 20% (BrS)–80% (LQTS) of all cardiac disorders listed above, depending on which disorder was being tested.

The aim was to design and validate a custom-cardiac panel that satisfied the diagnostic requirements of LabPLUS (Auckland City Hospital, Auckland, NZ), as well as the guidelines provided by the Royal College of Pathologists of Australasia (RCPA) [40,41,42] and the Association for Clinical Genetic Science (ACGS) [43]. The Agilent SureSelect^QXT^ Target Enrichment system (Agilent Technologies) was used to create the patient libraries for MPS. This system uses a hybridisation approach to enrich for target sequences of interest. Several different commercially available data analysis programs were also assessed to investigate which exhibited the highest sensitivity and specificity for variant calling and annotation of the custom-cardiac panel. A comparison of the performance of the custom-cardiac panel and a commercially available cardiac panel was also undertaken. The TruSight^®^ Cardio Sequencing kit (Illumina, San Diego, CA, USA) was chosen as it uses a hybridisation enrichment approach similar to the Agilent SureSelect^QXT^ Enrichment system. The Illumina kit targets 174 known genes associated with 17 heritable cardiac conditions; 28 of these genes were the same as those in our custom-designed cardiac panel.

## 2. Materials and Methods 

### 2.1. Samples

A total of 76 patients, who had previously been referred for cardiac gene mutation screening, were selected for the custom cardiac panel (SureSelect^QXT^ Target Enrichment System, Agilent Technologies, Santa Clara, CA, USA). Of these 76 samples, nine were negative for a causative gene variant and two of the 76 samples were chosen as inter-/intra-batch control samples. Some of the selected patient samples were extracted from post-mortem blood. Previous testing was performed for all 76 patient samples and testing was either performed by LabPLUS or The Churchill Hospital (Oxford, UK). The testing at LabPLUS involved Sanger-based sequencing of the coding regions of genes implicated in LQTS 1, 2, 3, 5, 6 and 7 (if requested), and the testing at The Churchill Hospital involved an MPS approach that comprised HCM, DCM and ARVC panels. Informed consent underpinned the diagnostic referrals.

A subset of the 76 patient samples (35 samples) were also analysed using the TruSight^®^ Cardio Sequencing kit (Illumina, San Diego, CA, USA). Of these 35 samples, two were negative for a causative gene variant and two were chosen as inter-/intra-batch control samples.

DNA samples extracted from living patients were obtained following informed consent under the approval of the New Zealand Multi-Regional Ethics Committee (AKX/02/00/107/AM2). In the case of deceased patients, the NZ National Coronial Service provided the authorisation to screen patient’s DNA for mutations in genes implicated in heritable cardiac disorders.

### 2.2. DNA Extraction

DNA was extracted from peripheral ethylene diamine tetra acetic acid (EDTA) blood samples using the Gentra^®^ Puregene^®^ Blood Kit (3 mL) (Qiagen, Venlo, Limburg, The Netherlands), according to manufacturer’s instructions. The quality and quantity of extracted genomic DNA (gDNA) were measured using a NanoDrop ND-1000 Spectrophotometer (Thermo Fisher Scientific, Waltham, MA, USA).

### 2.3. Custom Cardiac Panel Design (SureSelect^QXT^ Target Enrichment System)

The enrichment of 29 heritable cardiac genes (Table 1) was performed using a SureSelect^QXT^ Target Enrichment System (Agilent Technologies, Santa Clara, CA, USA). The cardiac panel underwent three design iterations. The first two iterations only contained 23 of the current 29 genes (*DSC2*, *DSG3*, *DSP*, *KCNJ2*, *PKP2* and *SCN10A* were not included). The number of probes and the capture size of each iteration are shown in Table 2. The number of samples processed and the number inter/-intra/ batch controls per iteration are also shown in Table 2.

### 2.4 Library Preparation and Sequencing 

#### 2.4.1. SureSelect^QXT^ Target Enrichment System

DNA samples were quantitated using a QuantiFluor^®^ double stranded DNA (dsDNA) system (Promega, Madison, WI, USA). The enrichment protocol was performed according to the manufacturer’s instructions (SureSelect^QXT^ Target Enrichment for Illumina Multiplexed Sequencing, version B.1, July 2014, Agilent Technologies) using approximately 50 ng of gDNA. Following enzymatic fragmentation, adaptor-tags were annealed to the fragmented DNA. The quality and quantity of each fragmented library was assessed using an Agilent TapeStation (Agilent Technologies) and D1000 ScreenTape. A peak DNA fragment size between 245–325 bp was expected. Hybridisation of the prepared gDNA library with the cardiac-specific capture library used 500–750 ng of the prepared gDNA. Once hybridised, the targeted molecules were captured using Dynabeads^®^ MyOne^™^ Streptavidin T1 (Invitrogen, Carlsbad, CA, USA). The cardiac-panel enriched DNA libraries were PCR amplified and sample-specific dual indices were applied. PCR products were purified using Agencourt^®^ AMPure^®^ XP beads (Beckman Coulter, Brea, CA, USA). The DNA quantity and quality of the enriched DNA libraries were assessed using an Agilent TapeStation, and a peak size between 325–450 bp was expected. Subsequent pooling of the enriched DNA libraries, comprising two batches of eight patient samples, was performed according to the manufacturer’s instructions. The library was submitted to New Zealand Genomics Limited (NZGL) for sequencing using an Illumina MiSeq^®^ platform. The pooled library was then further diluted to 8 pM and was sequenced on an Illumina MiSeq^®^ System using Illumina MiSeq^®^ Reagent Kit v2 (300 cycles series, 2 × 151 reads). The MiSeq^®^ MPS protocol was carried out according to the manufacturer’s instructions.

#### 2.4.2. TruSight^®^ Cardio Sequencing System

Samples were prepared in a batch size of 12 samples following the manufacturer’s instructions. Each sample was quantitated using a QuantiFluor^®^ dsDNA system (Promega). The enrichment protocol was performed according to the manufacturer’s instructions (TruSight^®^ Cardio Sequencing Panel Reference Guide, Part #15063774 Rev. A, March 2015, Illumina). An initial amount of 50 ng of DNA was used for optimal enrichment. After enzymatic tagmentation of each patient sample, the quality and quantity of the fragmented library was assessed on the Agilent TapeStation (Agilent Technologies) using the D1000 ScreenTape. A peak DNA fragment size between 300–1000 bp was expected. All patient samples were pooled to give a total of 6000 ng of DNA library. The pooled samples underwent two rounds of overnight hybridisation with the TruSight^®^ Cardio panel probes. Once hybridised, the targeted molecules were captured using streptavidin beads. The TruSight^®^ Cardio panel-enriched DNA libraries were PCR amplified and purified using magnetic purification beads provided by the kit. The DNA quantity and quality of the enriched DNA libraries were assessed using an Agilent TapeStation, and a peak size between 200–1000 bp was expected. The pooled library was further diluted to 8 pM and was subjected to sequencing using an Illumina MiSeq^®^ platform and Illumina MiSeq^®^ Reagent Kit v2 (300 cycles series, 2 × 151 reads). The MiSeq^®^ MPS protocol was carried out according to the manufacturer’s instructions.

### 2.5. Bidirectional Sanger-Based Sequencing

The appropriate gene exons containing the variants of interest were PCR amplified as follows: 1 × FastStart PCR buffer, 1 × GC-rich solution, 2 mM magnesium chloride, 0.8 µM each of the forward and reverse primer, 0.4 mM deoxynucleoside triphosphate (dNTP), 0.04 U FastStart Taq DNA Polymerase (Roche, Basel, Switzerland), and 50 ng of gDNA. The following cycling conditions were used: 95 °C for 4 min, 35 cycles of 94 °C for 45 s, 60 °C for 30 s, 72 °C for 2 min 45 s, and a final extension at 72 °C for 10 min. The PCR amplicons were visualised by electrophoresis in 2% E-gel^™^ EX Agarose Gels (Life Technologies, Camarillo, CA, USA). Bi-directional DNA sequencing was performed using BigDye^™^ Terminator v3.1 Cycle Sequencing (Applied Biosystems^™^ Ltd., Foster City, CA, USA). The sequenced products were purified using the BigDye XTerminator^™^ Purification Kit (Applied Biosystems^™^ Ltd.), and then were subjected to capillary electrophoresis using an Applied Biosystems^™^ model 3500xL Genetic Analyzer. The analysis of sequence traces was performed using Variant Reporter^®^ Software v2.0 (ThermoFisher Scientific, Cambridge, MA, USA) and Geneious v7.1.7 software (Biomatters, Auckland, New Zealand) [44].

### 2.6. Bioinformatic Processing 

Massively parallel sequencing reads were automatically processed for 3′ and 5′ adaptor trimming and FastQ files were generated. The minimal number of reads (minimum read-depth coverage) and sequencing error rates (also known as variant frequency (VF)) are the two chief parameters for accurate variant calling [45,46]. According to the RCPA guidelines, the minimum requirements for data quality should be determined by the diagnostic laboratory [40,41,42]. American College of Medical Genetics and Genomics (ACMG) has recommended a minimum mean read-depth of 30× for genome sequencing [47], and this threshold was used for the validation process. The sequencing error rate of 20% (per direction), corresponding to a Phred score equivalent of 33 [45,46], was set as the allelic fraction (variant frequency) in order to differentiate the true variants from false positive variant calls. This meant that a true variant should be present in at least 20% of forward and reverse reads in order to be detected as a variant by the analysis programmes used here.

#### 2.6.1. SureSelect^QXT^ Target Enrichment System

Raw sequence data were aligned using two different data analysis programmes (Agilent Technologies-SureCall and JSI Medical Systems GmbH-SeqPilot) against the appropriate Locus Reference Genomic (LRG) references (http://www.lrg-sequence.org/, last accessed June 2016) if they were available from the Human Genome assembly (HG19 build; Table 1). Locus Reference Genomic sequences are independent of updates to the reference genome assembly, and therefore provide a stable genomic DNA sequence for unambiguous reporting of gene variants. Human Genome Variation Society (HGVS) v2.0 nomenclature was used to describe all variants with nucleotide numbering starting from the first nucleotide of the translated sequence. The coding exons of the 29 cardiac genes, together with the 3–20 bp of flanking Intronic sequence, were analysed using both SureCall (Agilent Technologies) and SeqPilot softwares (JSI medical systems GmbH, New York, NY, USA).

#### 2.6.2. TruSight Cardio Sequencing System

Raw sequence data were aligned against the appropriate LRG reference sequences using SeqPilot software (JSI medical systems GmbH). The Variant Call Format (VCF) file generated from the Illumina MiSeq^®^ sequencing run was imported into the VariantStudio software (Illumina). The coding exons of the 29 cardiac genes, together with the 3–20 bp of flanking sequence, were analysed using the SeqPilot software (JSI medical systems GmbH) and VariantStudio software (Illumina).

### 2.7. Validation Process

#### 2.7.1. Read-Depth Analysis

The average read-depth of all exons in all genes of interest from both the custom-cardiac panel and TruSight^®^ Cardio Panel was calculated using the coverage tool from BEDTools [48]. The minimal number of reads (minimum coverage) and sequencing error rates (also known as variant frequency (VF)) were those stated earlier.

#### 2.7.2. Statistical Analysis

In accordance with the RCPA and ACGS guidelines the sensitivity, specificity, positive predictive value, and false discovery rate were calculated using each of the data analysis programmes [40,41,42,43]. Sensitivity is defined as the true positive rate, i.e., the percentage of correctly identified true variants in patients who have them. Sensitivity quantifies the avoidance of false negatives. It was considered necessary to demonstrate 95% confidence that the error rate for heterozygous/homozygous mutation detection was <5% [43]. This requires concordant results for a minimum of 60 unique variants tested by the MPS method compared to Sanger-based sequencing results [43,49]. Specificity is defined as the true negative rate, i.e., the correct identification of no variants in patients who have no variants. Specificity quantifies the avoidance of false positives. The positive predictive value (PPV) is defined as the probability that a base pair identified as a variant is truly a variant. Table 3 shows how true positive (TP), true negative (TN), false positive (FP) and false negative (FN) variants were defined and how the sensitivity, specificity and positive predictive value (PPV) were calculated.

#### 2.7.3. Inter/Intra-Run Repeatability Analysis

An inter-run repeatability analysis was carried out in the validation process in accordance with both the RCPA and ACMG guidelines [40,41,42,43]. Two of the 76 samples were selected as control samples. The variant calling for each replication was interrogated for result concordance.

## 3. Results

### 3.1. Library Preparation Workflow

**Figure 1:**
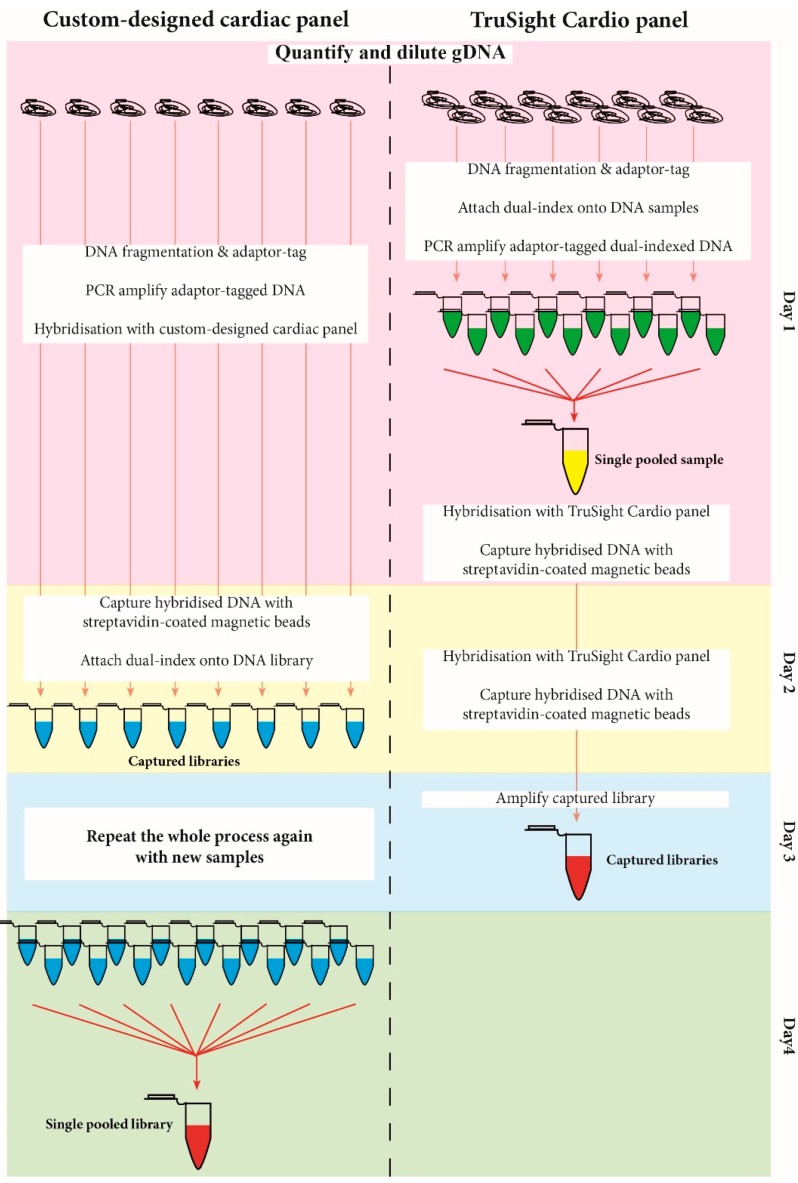
Comparison of workflows between custom-cardiac panel (Agilent SureSelect^QXT^ Enrichment system) and TruSight^®^ Cardio panel (Illumina).

The SureSelect^QXT^ and TruSight^®^ library preparation use baits that hybridise specifically to regions of interest and the bound bait-DNA library is captured using Streptavidin-bound magnetic beads. The custom-cardiac panel used the SureSelect^QXT^ capture system which multiplexes all samples post-capture (Figure 1). This means that each individual sample is hybridised and captured separately, and subsequently pooled for sequencing (Figure 1). Our approach described here comprised four days to capture and multiplex 2 × 8 samples for the custom-cardiac panel (in the case of processing 16 samples simultaneously, then this time commitment would be two days only). In addition, it offers more control over sample multiplexing (i.e., if a sample library amplified poorly, the sample volume used for multiplexing could be adjusted accordingly). The TruSight^®^ capture system multiplexes all samples before capturing (pre-capture). This involves labelling each DNA sample with unique barcodes and the samples are pooled before capturing occurs (Figure 1) [50]. Pre-capture multiplexing comprises three days to multiplex and capture 12 samples; however, there is less control over sample amplification efficiency [50].

### 3.2. Gene Read-Depth Analysis 

#### 3.2.1. Custom-Designed Cardiac Panel

The first two custom-cardiac panel designs contained only 23 of the 29 genes of the final design. These two iterations lacked the following genes: *DSC2*, *DSG2*, *DSP*, *KCNJ2*, *PKP2,* and *SCN10A*. All genes had full exon coverage, except for exon 186 of the *TTN* gene, which was not covered. The average read-depth was approximately 286×; however, six regions of interest had either a read-depth below 30× or were not 100% covered by the probes. These regions were *CACNB2* exon 2, *MYL2* exon 1, RYR2 exons 73, and *TTN* exons 10, 47, and 186 (Supplementary Figure S1). Additional probes were added to these six regions in the second design iteration.

The overall coverage of the genes in the second design iteration improved compared to the initial design. The average read-depth for iteration two was ~353×; however, there were still several regions that fell below the 30× read-depth criterion (Supplementary Figure S2) and the distribution of the read-depth for the second design iteration appeared to be more widely spread than the initial design (Supplementary Figure S2). It is not known why there was a difference in read-depth distribution between the two capture designs. The regions below 30× read-depth were *CACNB2* exon 2, *MYPBC3* exons 12, 22, *MYL2* exon 1, *SCN1B* exon 1, *RYR2* exons 5, 35, 54, 58, 96, and *TTN* exons 10, 47 and 186. Additional probes were added to these regions in the third design iteration to increase the read-depth. Six additional genes (*DSC2*, *DSG2*, *DSP*, *KCNJ2*, *PKP2* and *SCN10A*) were also included in the third and final iteration of the design. The additional genes expanded the disease repertoire of the panel.

The average read-depth for the third design iteration was ~268×. The distribution of read-depths for all exons is shown in Supplementary Figure S3. The read-depths for the majority of genes in the third design iteration were well above 30× (Supplementary Figure S3). There were only five regions that fell below the 30× threshold. These regions were: *MYL2* exon 1, *TTN* exons 10, 47, 178 and 189. It was decided to amplify these regions separately for subsequent Sanger-based sequencing.

The total number of reads of each sample pool for all three iterations of the Agilent designs, and the Illumina Cardio panel, are shown in Supplementary Data S2. The average number of reads for all samples in Agilent iteration 1 was approximately 986,000; iteration 2 was approximately 1,560,000; and iteration 3 was approximately 1,000,000.

#### 3.2.2. TruSight^®^ Cardio Panel

As the TruSight^®^ Cardio panel targets 174 genes, it was decided to analyse only the sequence data for those genes that were captured by the custom design cardiac panel (except for *SCN10A* which is not part of the TruSight^®^ Cardio panel). The average read-depth was approximately ~294×, and there were thirteen regions that fell below the 30× threshold (*GLA* exon 4, *KCNH2* exon 1 and 2; *KCNQ1* exon 5 and 15; *MYBPC3* exons 3, 11 and 26; *SCN1B* exon 1; *SCN5A* exon 3; *TNNI3* exon 1; and *TNNT2* exon 11 and 15; Supplementary Figure S4).

In the case of the Illumina Cardio panel, the average number of reads per sample pool is 2,640,000. It should be noted that the Illumina Cardio panel interrogates 174 genes compared to the 29 genes that are present in the custom-designed cardiac panel.

#### 3.2.3. Variant Calling Concordance

A wide range of variants from as many targeted genes as possible was chosen. A list of the variants tested is shown in Supplementary Data S1, and a summary of the types of variants tested (i.e., missense, deletion, etc.) for each gene is shown in Table 4.

#### 3.2.4. Custom-Designed Cardiac Panel

Sixteen patient samples were captured using the first design iteration of the custom-cardiac panel. There were a total of 171 expected variants (true positives–TPs), most of which were missense variants (Table 5). The majority of the variants were located in the *SCN5A*, *KCNH2* and *RYR2* genes (Table 6). A summary of the results for all three capture design iterations for the custom-cardiac panel is presented in Table 6. JSI SeqPilot analysis outcomes were concordant for all expected TPs, while SureCall was concordant for 170 expected TPs (Table 7). The variant that SureCall could not accurately detect was in the *MYBPC3* gene (*MYBPC3* c.158_160delACAinsTGGTCACAG). The software was able to detect the deletion event in the correct location; however, it was unable to correctly annotate the insertion event. SureCall was discordant with Sanger-base sequencing data for one variant (false positive—FP), while JSI SeqPilot was discordant for five variants. These FPs are associated with deletions/duplications in homopolymeric runs of 9–11 bp from the exonic regions of *CASQ2* and *RYR2*. The sensitivity, specificity and positive predictive value (PPV) were all 99.42% using SureCall software. In the case of JSI SeqPilot software, sensitivity was 100%, and specificity and PPV were both 97.16% (Table 7).

The second design iteration involved processing 32 patient samples and a total of 420 expected TPs (Table 2 and Table 5). The majority of variants were missense (Table 5), most of which were located in the *TTN* gene (Table 6). SureCall analysis outcomes were concordant for all expected TPs and JSI SeqPilot was concordant for 419 TPs (Table 7). The variant that JSI SeqPilot was not able to detect was in the *TTN* gene (*TTN* c.10256G > A). Both SureCall and JSI SeqPilot detected the same two FPs found in two different patient samples (*CASQ2* c.738-5_8del). This is a homopolymeric region containing 23 As. The sensitivity for SureCall was 100% and the specificity and PPV were 99.53% (Table 7). The sensitivity for JSI SeqPilot was 99.76% and the specificity and PPV were 99.53% (Table 7).

A total of 48 patient samples were processed using the third iteration design (Table 2). The expected number of TPs was 587 variants with a strong representation of missense and intronic variants (Table 5). The majority of these variants were localised to the *MYH7*, *SCN5A*, *TTN*, *KCNH2* and *MYBPC3* genes (Table 6). Both SureCall and JSI SeqPilot analysis outcomes were concordant and detected two FPs (Table 7). The two FPs were from different patient samples and both software programmes detected the same FPs. One FP was located in the *PKP2* gene (*PKP2* c.1759G > A), which could not be confirmed using Sanger-based sequencing. The other FP was located in the *MYL2* gene (*MYL2* c.274+16_17insCT). This is a dinucleotide repeat region containing nine CTs. The sensitivity of both SureCall and JSI SeqPilot was 100%, and the specificity and PPV were 99.66% for both software programmes (Table 7).

Following the ACGS guidelines, 63 distinct variants within the *DSC2*, *DSG2*, *DSP*, *KCNE1*, *KCNE2*, *KCNH2*, *KCNJ2*, *KCNQ1*, *SCN1B* and *SCN5A* genes were analysed and compared to previous Sanger sequence data (Supplementary Data S1). Full concordance was observed for the 63 distinct gene variants (from 27 patient samples) analysed by both software programmes (SureCall and JSI SeqPilot). These patient samples were taken from all three design iterations as the LQTS genes had not changed in design across all three iterations.

#### 3.2.5. TruSight^®^ Cardio Panel

A total of 35 patient samples were processed using the TruSight^®^ Cardio panel, and a total of 584 expected TPs (Table 5). These 35 samples were selected from the pool of samples that had been processed using the custom-cardiac panel. A summary of the results for the TruSight^®^ Cardio panel is shown in Table 7. VariantStudio analysis outcomes were concordant for 472 of the 584 expected TPs, while JSI SeqPilot was concordant for 580 expected TPs (Table 8). The majority of discordant variants using VariantStudio were intronic variants, insertions/duplications and deletions (Supplementary Data S2). VariantStudio could not detect any of the 78 intronic variants, and only called 4 of 10 insertions/duplications, and 8 of the 36 deletions (Supplementary Data S2). The majority of the insertions/duplications and deletions were located to intronic regions (i.e., *MYBPC3* c.506-12delG, *MYL2* c.353+20delC, *TNNT2* c.53-7_11delAGAAG). There were also some deletion/duplication events that VariantStudio could not identify, for example: *KCNH2* c.221_242del, c.1872_1882dup, *MYBPC3* c.158_160delACAinsTGGTCACAG, c.2864_2865delCT). The five variants that JSI SeqPilot was discordant for were *DSC2* c.1dupA, *MYBPC3* c.2864_2865delCT, *SCN5A* c.2788-6C > T and *SCN1B* c.40+15G > T. The sensitivity, specificity and PPV for VariantStudio were 80.82%, 99.58% and 99.58%, respectively (Table 8). For JSI SeqPilot, the sensitivity, specificity and PPV were 99.14%, 99.15% and 99.15%, respectively (Table 8).

The results from the same patient samples processed using the custom-cardiac panel and analysed using both SureCall and JSI SeqPilot software programmes were compared to the TruSight^®^ Cardio panel data. The results showed that SureCall was discordant for only one variant (*MYBPC3* c.158_160delACAinsTGGTCACAG, Table 8), while JSI SeqPilot was discordant for three variants (Table 8). These were *KCNH2* c.1539C > T, *MYBPC3* c.2864_2865delCT and *TTN* c.10256G > A. The sensitivity, specificity and PPV for SureCall for this set of samples were 99.83%, 99.32% and 99.32%, respectively (Table 8). For JSI SeqPilot, the sensitivity, specificity and PPV were 99.49%, 98.98%, and 98.98%, respectively (Table 8).

#### 3.2.6. Inter/Intra-Run Repeatability Analysis

Two samples were included as inter/intra-run repeatability samples. Both samples showed 100% concordance between/within replicates for all runs for the second and third design iterations of the custom-cardiac panel, and also for the TruSight^®^ Cardio panel.

## 4. Discussion

Here we describe the process that was undertaken to design and validate a custom-cardiac panel suitable for a diagnostic environment. We also compared the performance of a custom-cardiac panel against a commercially-available pre-designed cardiac panel to evaluate the differences between the panels in terms of library preparation workflow and data quality. All data analysis was performed using three commercially available software programmes, two of which (SureCall and VariantStudio) were designed specifically to analyse data from the two different capture systems (Agilent and Illumina) that are reported here. The third software programme (JSI SeqPilot) was already in use in the Diagnostic Genetics Department (LabPLUS, Auckland City Hospital, NZ) as a means of identifying *BRCA1/2* gene variants. These software programmes were compared and evaluated in terms of their overall performance.

The overall library preparation workflow between the custom-cardiac panel and the TruSight^®^ Cardio panel differed in the multiplexing steps (post-multiplex and pre-multiplex, respectively), which affected the hands-on processing time (pre-multiplexing is less labour-intensive). However, both panels took about the same amount of time to prepare for sequencing (three days for TruSight^®^ and four days for the custom-cardiac panel). The custom cardiac panel could multiplex more samples (16 compared to 12) and offered more control over the amount of patient library pooled in the multiplexing step. Table 9 summarises the differences between the two panels.

For the final custom-cardiac panel, both SureCall and JSI SeqPilot correctly called and annotated all variants that were tested and both programs made two FP calls. While there were several regions that required Sanger-based sequencing to ensure complete coverage of two of the targeted genes, the custom-cardiac panel provided everything that was needed for a small diagnostic laboratory offering cardiac genetic testing. There was also room to increase the repertoire of cardiac genes in the custom-cardiac panel should the need arise. The sensitivity, specificity and PPV of both programmes showed that the custom-cardiac panel could pair with either programme to correctly call and annotate variants to a high standard, which adheres to the guidelines that were chosen for the validation process [40,41,42,43].

The TruSight^®^ Cardio panel targets 174 cardiac-related genes; however, only those genes that corresponded with the custom-cardiac panel were analysed. JSI SeqPilot performed better than VariantStudio in calling and annotating the variants that were tested; however, JSI SeqPilot also made more FP calls. Overall, JSI SeqPilot was the better program to use to analyse the TruSight^®^ Cardio panel results. The sensitivity, specificity and PPV for JSI SeqPilot were comparable to the results obtained by the custom-cardiac panel; however, the TruSight^®^ Cardio panel had more regions that required Sanger-based sequencing compared to the custom-cardiac panel (Table 8).

While the TruSight^®^ Cardio panel targeted many more genes than our custom-cardiac panel, the number of regions that required Sanger-based sequencing to ensure all genes were completely covered increased the amount of labour required per patient sample. For a small diagnostic laboratory, the chosen MPS approach should target the genes of interest and generate high quality and high read-depth sequencing data that minimises the extent of Sanger-based sequencing “fill-in”. In this case, the custom-cardiac panel satisfied this despite targeting fewer genes.

From the clinical perspective there is a need to balance the high probability of detection of a malignant mutation against finding large numbers of variants of uncertain significance in genes of unlikely correlation to the clinical phenotype. There is no doubt that such findings can add to the anxiety and uncertainty already faced by families in this situation, which has been described as “genetic purgatory” [51]. Recent research into the molecular autopsy reveals vast numbers of variants of uncertain significance [5]. This research is essential but our aim is first to provide a valuable clinical service while minimising the risk of putting our families into such purgatory and minimising clinical bioinformatic and genetic counselling workloads.

## 5. Limitations

With the advance in genetic testing of heritable cardiac diseases, it is still important to accurately assess a patient’s family history and marry clinical results with genetic results. While the ability to interrogate multiple genes of a single patient simultaneously is a significant step forward in genetic testing, the results can be complicated if they are not accurately interpreted. A bottleneck of diagnostic MPS testing is the sheer volume of data it produces. It is not feasible to functionally test all likely pathogenic variants in a diagnostic environment, and in many diagnostic laboratories the pathogenicity of all gene variants are determined using in silico prediction programs. In our own laboratory, we have found that the use of these programs is gene specific, and some programs may work better together than others [52]. However, as these are only prediction programs, the ultimate test to verify a variant’s pathogenicity is either via functional studies or thorough familial segregation analysis.

Currently, the genes chosen for the custom-cardiac panel cover 20% (BrS)–80% (LQTS) of the diagnosis of each of the syndromes listed in Table 1. Therefore, a negative result does not necessarily mean the disorder has been excluded in the diagnosis. These negative results could be due to the lack of sensitivity of the test or the region where the mutation lies outside of the tested region. Therefore, the custom-cardiac panel should act as a first tier genetic test. If a phenotypic-positive patient returns with a negative result from the custom-cardiac panel their samples should be further tested using whole exome/genome sequencing, ideally as part of a registry-based translational research project.

## 6. Conclusions

The approach described here had a large development cost associated with it, but had the advantage of offering control in terms of focusing on a gene list-of-choice to satisfy the clinical demands and achieve (for the most part) the quality control metrics associated with MPS. In contrast, accessing a commercially-validated test comprising a large gene panel offers a clinical laboratory the means to short-circuit any investment in test development although at a cost in terms of sequencing genes that are of modest clinical impact (in the context of detecting a pathogenic mutation) as well as the likelihood of less-than-adequate read depths for all coding regions. Critically, this “tension” between a custom-design focused gene panel and a large panel/clinical exome approach must be addressed by each clinical laboratory based on resources (both equipment and staffing), batch sizes, and turnaround times. In the context of heritable cardiac disorders, we have taken the view that our referrals are compatible with a focused gene panel approach.

## Figures and Tables

**Table 1:** List of cardiac genes included in the custom gene panel.

Gene	Transcript Accession #	LRG Reference #	ARVC	BrS	CPVT	DCM	HCM	LQTS	SQTS
*BAG3*	NM_004281.3	LRG_742t1							
						2–4% [13]			
*CACNA1C*	NM_000719.6	LRG_334t1		BrS 3					SQT 4
				2–12% [14,15,16]					Limited data [17]
*CACNB2*	NM_201596.2	LRG_381t1		BrS 4					SQT 5
		(pending approval)		2–12% [14,15,16]					Limited data [17]
*CASQ2*	NM_001232.3	LRG_404t1			CPVT 2				
					Rare [18]				
*DSC2*	NM_004949.3	LRG_400t1	ARVC 11						
			2–7% [19]						
*DSG2*	NM_001943.3	LRG_397t1	ARVC 10						
			7–26% [20,21,22]						
*DSP*	NM_004415.2	LRG_423t1	ARVC 8						
			6–16% [23]			1–3% [13]			
*GLA*	NM_000169.2	LRG_672t1							
							0.5–3% [24,25]		
*KCNA5*	NM_002234.2								
									
*KCNE1*	NM_000219.3	LRG_290t1						LQT 5	
								<1% [26]	
*KCNE2*	NM_172201.1	LRG_291t1						LQT 6	
								<1% [26]	
*KCNH2*	NM_000238.3	LRG_288t1						LQT 2	SQT 1
								35–45% [26]	Limited data [17]
*KCNJ2*	NM_000891.2	LRG_328t1						LQT 7	SQT 3
								<1% [26]	Limited data [17]
*KCNQ1*	NM_000218.2	LRG_287t1						LQT 1	SQT 2
								40–55% [26]	Limited data [17]
*LMNA*	NM_005572.3	LRG_254t1							
						4–8% [13]			
*MYBPC3*	NM_000256.3	LRG_386t1							
							>20% [27]		
*MYH6*	NM_002471.	LRG_389t1							
							Rare [27]		
*MYH7*	NM_000257.2	LRG_384t1							
						4–8% [13]	>30% [27]		
*MYL2*	NM_000432.3	LRG_393t1							
							<5% [27]		
*PKP2*	NM_004572.3	LRG_398t1	ARVC 9						
			[28,29]						
*RBM20*	NM_001134363.1	LRG_382t1							
						3–6% [13]			
*RYR2*	NM_001035.2	LRG_402t1	ARVC 2		CPVT 1				
		(pending approval)	Rare [30]		50% [31]				
*SCN1B*	NM_001037.4	LRG_420t1		BrS 5					
		(pending approval)		<1% [14]					
*SCN5A*	NM_198056.2	LRG_289t1		BrS 1				LQT 3	
				2–30% [32]		1–2% [13]		2–8% [26]	
*SCN10A*	NM_006514.3			BrS 21					
				[33,34]					
*TNNI3*	NM_000363.4	LRG_432t1							
							>5% [27]		
*TNNT2*	NM_000364.2	LRG_431t1							
		(pending approval)				3–6% [13]	>20% [27]		
*TPM1*	NM_001018005.1	LRG_387t1							
		(pending approval)				2–4% [13]	>5% [27]		
*TTN*	NM_001267550.1	LRG_391t1							
						15–25% [13]	Rare [35]		
**% of diagnosis covered**			**45–70% [36]**	**20–25% [37]**	**50–70%**	**20–25% [36]**	**60% [36]**	**75–80%**	**NA**
					**[36,37,38,39]**			**[36,37,38,39]**	

LRG: Locus reference genomic; ARVC: arrhythmogenic right ventricular cardiomyopathy; BrS: Brugada Syndrome; CPVT: catecholaminergic polymorphic ventricular tachycardia; DCM: dilated cardiomyopathy; HCM: hypertrophic cardiomyopathy; LQTS: long QT syndrome; SQTS: short QT syndrome.

**Table 2:** Number of samples processed per iteration for the custom-designed cardiac panel.

	Custom-Designed Cardiac Panel	TruSight Cardio Panel		
	Iteration 1	Iteration 2	Iteration 3	
**Number of probes**	9556	14,038	15,123	N/A
**Capture size (kbp)**	293.727	307.071	344.742	572.00
**Number of individual samples**	16	24	36	31
**Number of inter/intra-run controls**	0	8	12	4
**Total number of samples**	16	32	48	35

**Table 3:** Definition of true negative (TN), false positive (FP) and false negative (FN) variants, as well as sensitivity, specificity and positive predictive value (PPV) formulae.

		Sanger-Based Results		
		Variant Detected	Variant not Detected	
**MPS results**	**Variant detected**	TP	FP	PPV
				[TP/(TP + FP)] × 100%
	**Variant not detected**	FN	TN	
		Sensitivity	Specificity	
		[TP/(TP + FN)] × 100%	[TN/(FP + TN)] × 100%	

MPS: Massively parallel sequencing.

**Table 4:** Number of unique variants tested for each gene.

Gene	Missense	Deletion	Insertion/Duplication	Intronic	Total
***BAG3***					
***CACNA1C***					
***CACNB2***					
***CASQ2***		2		3	5
***DSC2***			1	2	3
***DSG2***		1		6	7
***DSP***			1	10	11
***GLA***		2			2
***KCNA5***					
***KCNE1***				3	3
***KCNE2***					
***KCNH2***	4		3	10	17
***KCNJ2***				4	4
***KCNQ1***	4	6	1	7	18
***LMNA***	1			4	5
***MYBPC3***	4	4	2	12	22
***MYH6***					
***MYH7***		1	1	18	20
***MYL2***	2			1	3
***PKP2***				5	5
***RBM20***					
***RYR2***		8		11	19
***SCN1B***					
***SCN5A***		1		1	2
***SCN10A***	1	5	1	12	19
***TNNI3***		3		2	5
***TNNT2***	2	2		7	11
***TPM1***				4	4
***TTN***		19		96	115
**Total**	**18**	**54**	**10**	**218**	**300**

**Table 5:** Total number of expected variants, and the breakdown of the number of variants that are missense, deletion, insertion/duplication and intronic variants for each iteration of the custom-cardiac panel, and TruSight^®^ Cardio panel.

Iteration	Total # Variants Expected	# Missense Variants	# Deletion Variants	# Insertion/Duplication Variants	# Intronic Variants
1	171	132	9	1	29
2	420	323	27	4	66
3	587	469	38	10	70
**Total**	**1178**	**924**	**74**	**15**	**165**
**TruSight**	**584**	**460**	**36**	**10**	**78**

**Table 6:** Number of variants tested for each gene for each iteration for the custom-cardiac panel and TruSight^®^ Cardio panel.

Gene	Iteration 1	Iteration 2	Iteration 3	Total	TruSight
***BAG3***					
***CACNA1C***					
***CACNB2***					
***CASQ2***	4	6		**10**	**6**
***DSC2***			6	**6**	**4**
***DSG2***			15	**15**	**9**
***DSP***			29	**29**	**26**
***GLA***			2	**2**	**2**
***KCNA5***					
***KCNE1***	10	10	16	**36**	**11**
***KCNE2***					
***KCNH2***	39	28	63	**130**	**39**
***KCNJ2***			6	**6**	
***KCNQ1***	15	13	14	**42**	**16**
***LMNA***		5	5	**10**	**10**
***MYBPC3***	8	36	56	**100**	**43**
***MYH6***					
***MYH7***	9	44	93	**146**	**56**
***MYL2***		4	3	**7**	**5**
***PKP2***			5	**5**	**3**
***RBM20***					
***RYR2***	25	31	1	**57**	**31**
***SCN1B***	2		2	**4**	**4**
***SCN5A***	48	61	78	**187**	**72**
***SCN10A***					
***TNNI3***	4	23	45	**72**	**26**
***TNNT2***	4	27	44	**75**	**29**
***TPM1***	3	15	27	**45**	**15**
***TTN***		117	77	**194**	**176**
**Total**	**171**	**420**	**587**	**1178**	**583**

**Table 7:** Summary of results for custom-cardiac panel analysed using SureCall and JSI SeqPilot.

	Iteration 1		Iteration 2		Iteration 3	
	SC	JSI	SC	JSI	SC	JSI
**Expected TP**	171		420		587	
**TP detected**	170	171	420	419	587	587
**FP detected**	1	5	2	2	2	2
**Sensitivity**	99.42%	100%	100%	99.76%	100%	100%
	(96.78–99.99%)	(96.78–99.99%)	(99.13–100.00%)	(98.69–99.99%)	(99.37–100.00%)	(99.37–100.00%)
**Specificity**	99.42%	97.16%	99.53%	99.53%	99.66%	99.66%
	(97.26–100.00%)	(93.46–99.07%)	(98.30–99.94%)	(98.30–99.94%)	(98.78–99.96%)	(98.78–99.96%)
**Positive predictive value**	99.42%	97.16%	99.53%	99.52%	99.66%	99.66%
	(96.78–99.99%)	(93.50–99.07%)	(98.30–99.94%)	(98.30–99.94%)	(98.78–99.96%)	(98.78–99.96%)

SC, SureCall.

**Table 8:** Summary of results for TruSight^®^ Cardio panel analysed using VariantStudio and JSI SeqPilot, and the same sample results using custom-cardiac panel analysed using SureCall and JSI SeqPilot.

	TruSight Cardio Panel		Custom-Designed Cardiac Panel	
	VariantStudio	JSI	SureCall	JSI
**Expected TP**	584		584	
**TP detected**	472	580	583	581
**FP detected**	2	5	4	6
**Sensitivity**	80.82%	99.14%	99.83%	99.49%
	(77.39–83.94%)	(98.26–99.81%)	(99.05–100.00%)	(98.51–99.89%
**Specificity**	99.58%	99.15%	99.32%	98.98%
	(98.48–99.95%)	(98.02–99.72%)	(98.26–99.81%)	(97.78–99.62%)
**Positive predictive value**	99.58%	99.15%	99.32%	98.97%
	(98.48–99.95%)	(98.02–99.72%)	(99.05–100.00%)	(97.78–99.62%)

**Table 9:** Comparison between the custom-cardiac panel and TruSight^®^ Cardio panel.

Design		
	Custom-Cardiac Panel	TruSight^®^ Cardio Panel
Number of cardiac genes targeted	29	174
		(*SCN10A* is not part of this panel)
**Workflow**		
**Number samples processed**	8 samples per working batch	12 samples for working batch
	16 samples for a full MiSeq run	12 samples for a full MiSeq run
**Time for library preparation (including incubation time)**	2 days for 8 samples (could be scaled to 16 samples for the same time)	3 days for 12 samples
**Capture workflow type**	Post-capture pooling	Pre-capture pooling
**Labour intensity**	More labour intensive than pre-capture pooling	Less labour intensive than post-capture pooling
**Control over quality of sample libraries**	More control over each sample library	Less control over sample libraries
**Coverage**		
**Regions with poor read-depth (<30×)**	*MYL2* exon 1	*GLA* exon 4
	*TTN* exon 10, 47, 178 and 189	*KCNH2* exon 1 and 2
		*KCNQ1* exon 5 and 15
		*MYBPC3* exon 3, 11 and 26
		*SCN1B* exon 1
		*SCN5A* exon 3
		*TNNI3* exon 1
		*TNNT2* exon 11 and 15
	**Total # regions: 5**	**Total # regions: 13**

## Supplementary Materials

The following are available online at www.mdpi.com/2076-3271/5/4/22/s1, Figure S1: title, Table S1: title, Video S1: title. Supplementary data S1: List of the variants tested; Supplementary data S2: Breakdown of the variant calls made by SureCall, JSI SeqPilot and VariantStudio for data generated by the custom-cardiac panel and TruSight^®^ Cardio panel; Supplementary Figure S1: Read-depth (coverage) of all exons for all genes from iteration one of the custom-cardiac panel. The dashed red line indicates the 30× read-depth threshold; Supplementary Figure S2: Read-depth (coverage) of all exons for all genes from iteration two of the custom-cardiac panel. The dashed red line indicates the 30× read-depth threshold; Supplementary Figure S3: Read-depth (coverage) of all exons for all genes from iteration three of the custom-cardiac panel. The dashed red line indicates the 30× read-depth threshold; Supplementary Figure S4: Read-depth (coverage) of all exons for all genes that were analysed from the TruSight^®^ Cardio panel. The dashed red line indicates the 30× read-depth threshold.
