# Supplementary material for: Massively Parallel Sequencing of Genes Implicated in Heritable Cardiac Disorders: A Strategy for a Small Diagnostic Laboratory
Source: Med Sci (Basel). 2017 Oct 10;5(4):22. doi: 10.3390/medsci5040022 (PMC5753651; doi:10.3390/medsci5040022)
Supplement: Supplementary file 1 [file medsci-05-00022-s001.zip › Supplementary data 1.pdf]

Gene	Variant	Frequency
CASQ2	c.196 A>G	1
	c.420+6 T>C	3
	c.784-17 T>A	2
	c.1185 C>T	3
	c.1194T>C	1
DSC2	c.111A>G	1
	c.942+12_13insTTA	3
	c.2393G>A	2
DSG2	c.828+16C>A	3
	c.861C>T	3
	c.877A>G	1
	c.2137G>A	3
	c.2318G>A	1
	c.2505A>G	1
	c.3321T>C	3
DSP	c.1dupA	2
	c.126T>C	1
	c.741T>G	2
	c.2091A>G	5
	c.2631G>A	5
	c.2862C>T	2
	c.4117A>G	1
	c.5213G>A	1
	c.7122C>T	4
	c.8175C>A	1
	c.8472G>C	5
GLA	c.-10C>T	1
	c.640-16A>G	1
KCNE1	c.84G>A	1
	c.112A>G	34
	c.253G>A	1
KCNH2	c.125T>A	1
	c.221_242del	1
	c.456dupC	1
	c.571insCTGCTG	1
	c.1467C>T	19
	c.1504A>C	1
	c.1539C>T	19
	c.1682C>T	12
	c.1692A>G	31
	c.1843C>G	1
	c.1872_1882dup	1
	c.1956T>C	34
	c.2230C>T	1
	c.2246delG	1
	c.2690A>C	4
	c.2886delG	1
	c.3093_3106del	1
KCNJ2	c.244C>T	1
	c.935G>A	1
	c.1146C>T	3
	c.1229A>G	1

KCNQ1	c.477+5G>A	1
	c.478-10G>A	1
	c.567delG	1
	c.572_576del	1
	c.727C>T	1
	c.733_734del	1
	c.797T>C	1
	c.805G>A	1
	c.998_999delCT	1
	c.1024C>T	1
	c.1033-2A>G	1
	c.1066C>T	1
	c.1090_1095dup	1
	c.1349-1G>T	1
	c.1394-14C>T	2
	c.1590+14T>C	1
	c.1638G>A	18
	c.1986C>T	7
LMNA	c.175C>A	1
	c.1017G>A	1
	c.1046G>A	1
	c.1304_1307delGCAC	2
	c.1698C>T	5
MYBPC3	c.158_160delACAinsTGGTCACAG	1
	c.472G>A	6
	c.492C>T	1
	c.506-12delG	23
	c.537C>T	1
	c.596T>G	1
	c.649A>G	1
	c.706A>G	14
	c.786C>T	13
	c.1286C>T	1
	c.1484G>A	1
	c.1624G>C	1
	c.1624+4A>T	1
	c.1855G>A	1
	c.2308+18C>G	1
	c.2373dupG	2
	c.2547C>T	1
	c.2737+12C>T	1
	c.2827C>T	1
	c.2864_2865delCT	1
	c.3617delG	1
	c.3288G>A	25
MYH7	c.77C>T	1
	c.189T>C	29
	c.732C>T	18
	c.597A>G	2
	c.895+17G>A	1
	c.925G>A	1
	c.975C>T	1
	c.1062C>T	4

	c.1063G>T	1
	c.1095G>A	15
	c.1128C>T	15
	c.2360G>A	1
	c.2389G>A	1
	c.2681A>G	11
	c.2967T>C	25
	c.3064A>G	1
	c.3337-2_3insC	2
	c.4472C>G	1
	c.5106G>A	12
	c.5172C>G	1
MYL2	c.132T>C	2
	c.353+20delC	3
	c.485_487delGAG	1
PKP2	c.209G>T	1
	c.419C>T	1
	c.1097T>C	1
	c.1759G>A	1
	c.2058T>A	1
RYR2	c.464-8A>C	3
	c.677-11T>A	4
	c.1259G>A	1
	c.1359C>T	4
	c.1612+14T>C	3
	c.1847C>T	1
	c.2973A>G	4
	c.6737C>T	1
	c.6906T>C	4
	c.7806C>T	3
	c.8873A>G	2
	c.9318T>G	4
	c.10503C>T	4
	c.10776C>T	4
	c.10935+18C>T	4
	c.11963-11T>C	1
	c.13476+16A>G	2
	c.13783-6A>G	4
	c.13913+12A>C	4
SCN1B	c.40+15G>T	2
	c.629T>C	2
SCN5A	c.87A>G	41
	c.612-2A>G	1
	c.1141-3C>A	22
	c.1673A>G	26
	c.2436+12G>A	12
	c.2788-6C>T	4
	c.3032C>T	1
	c.3183A>G	44
	c.3269C>T	1
	c.3308C>A	1
	c.3510+10C>T	1
	c.3572G>A	1

	c.3578G>A	6
	c.4218G>A	1
	c.4231delG	1
	c.4848C>T	1
	c.5350G>A	1
	c.5385_5387dupTGA	1
	c.5457T>C	21
TNNI3	c.25-8T>A	20
	c.109-17C>A	2
	c.204G>T	1
	c.373-10T>G	35
	537G>A	14
TNNT2	c.52+7G>A	1
	c.53-7_11delCTTCT	32
	c.113C>T	1
	c.144C>T	1
	c.237G>A	2
	c.311G>T	1
	c.348C>T	33
	c.418C>T	1
	c.517_519delGAG	1
	c.601-1G>A	1
	c.779A>G	1
TPM1	c.453C>A	32
	c.486T>C	11
	c.574G>A	1
	c.688G>A	1
TTN	c.982C>T	1
	c.2244G>A	2
	c.2432C>T	5
	c.3087T>C	1
	c.3601A>G	7
	c.3759A>G	1
	c.38034T>C	1
	c.3884C>T	7
	c.4480+6C>T	7
	c.4715G>A	1
	c.5132C>T	1
	c.7545C>T	2
	c.7830G>C	1
	c.8492G>A	1
	c.8902+14T>A	1
	c.9597A>G	1
	c.9781G>A	7
	c.9879A>G	7
	c.10256G>A	7
	c.10793G>A	1
	c.10878C>T	7
	c.13811G>A	1
	c.14297-19G>C	1
	c.16086A>G	1
	c.18652G>C	1
	c.19367-3T>C	1

c.19491G>A	7
c.20699A>C	1
c.20784C>T	2
c.21241A>G	1
c.21332C>A	2
c.21542G>A	1
c.21894G>T	1
c.21975T>C	1
c.22359A>T	2
c.22557A>G	2
c.22676A>G	1
c.22923C>T	2
c.24930G>A	1
c.26031T>C	1
c.26067G>A	1
c.27079A>G	1
c.27832A>G	2
c.28132G>A	2
c.28916G>A	1
c.29075-10T>A	1
c.29555G>A	2
c.30102G>A	1
c.33804T>C	1
c.34367A>G	1
c.35254A>G	2
c.35315T>C	1
c.42110T>G	1
c.42281A>C	1
c.43980G>A	1
c.45117T>C	1
c.45419A>T	1
c.46387A>G	1
c.46485T>C	1
c.48397A>G	2
c.49611T>C	2
c.50732G>A	1
c.51229C>T	1
c.51881C>T	1
c.52434T>C	1
c.53541A>G	2
c.53791C>T	1
c.54354T>C	2
c.56504C>T	2
c.56572C>T	1
c.57871+19T>G	1
c.57978A>G	2
c.58025T>C	1
c.58910G>A	1
c.59371G>A	1
c.59395T>C	1
c.59542G>C	7
c.59931T>C	1
c.59933-17T>C	1

	c.63126C>T	2
	c.67135C>T	1
	c.69016T>C	1
	c.69575A>G	1
	c.69934A>G	1
	c.70970T>C	2
	c.71561T>C	1
	c.72158C>T	1
	c.73095C>A	1
	c.74151C>T	1
	c.75619A>G	2
	c.75969T>C	2
	c.79383T>C	1
	c.80483T>C	2
	c.84148+8T>A	1
	c.84487A>G	1
	c.85539C>T	1
	c.87343A>G	1
	c.89909G>A	1
	c.90091+6G>T	2
	c.90243G>C	1
	c.90394+9T>A	1
	c.90891A>G	1
	c.91327T>A	1
	c.91730G>A	1
	c.92392G>A	1
	c.93062-10T>C	1
	c.94815C>T	1
	c.96077G>A	1
	c.97284C>T	1
	c.97680A>G	2
	c.98571G>C	1
	c.98854G>A	1

Supplementary data 1: List of the variants tested.

Variants highlighted in yellow make up the list of unique variants required to satisfy the ACGS guidelines.
