# Supplementary material for: Massively Parallel Sequencing of Genes Implicated in Heritable Cardiac Disorders: A Strategy for a Small Diagnostic Laboratory
Source: Med Sci (Basel). 2017 Oct 10;5(4):22. doi: 10.3390/medsci5040022 (PMC5753651; doi:10.3390/medsci5040022)
Supplement: Supplementary file 1 [file medsci-05-00022-s001.zip › Supplementary figures.pdf]

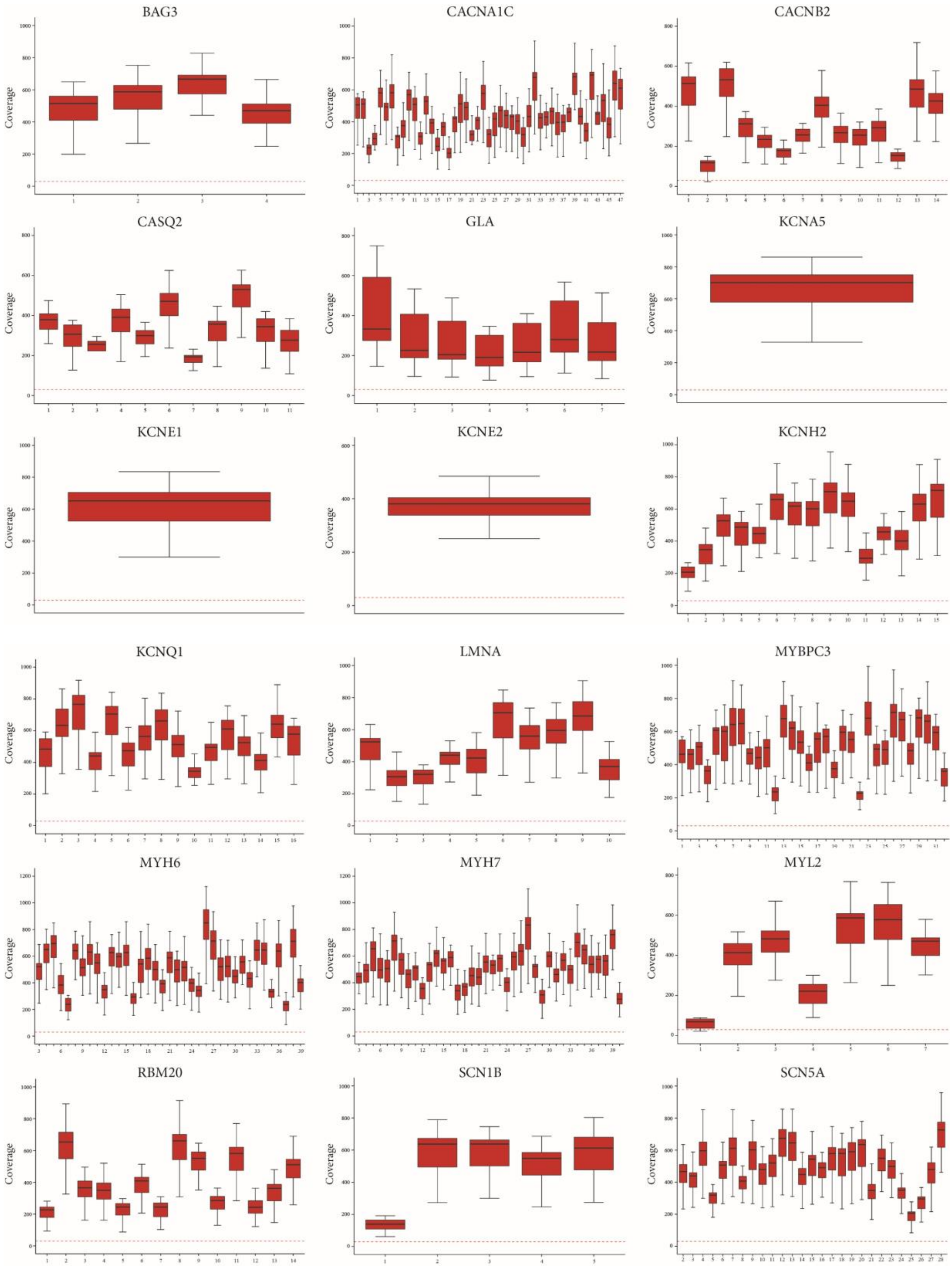
BAG3
CACNA1C
CACNB2
CASQ2
GLA
KCNA5
KCNE1
KCNE2
KCNH2
KCNQ1
LMNA
MYBPC3
MYH6
MYH7
MYL2
RBM20
SCN1B
SCN5A
Coverage

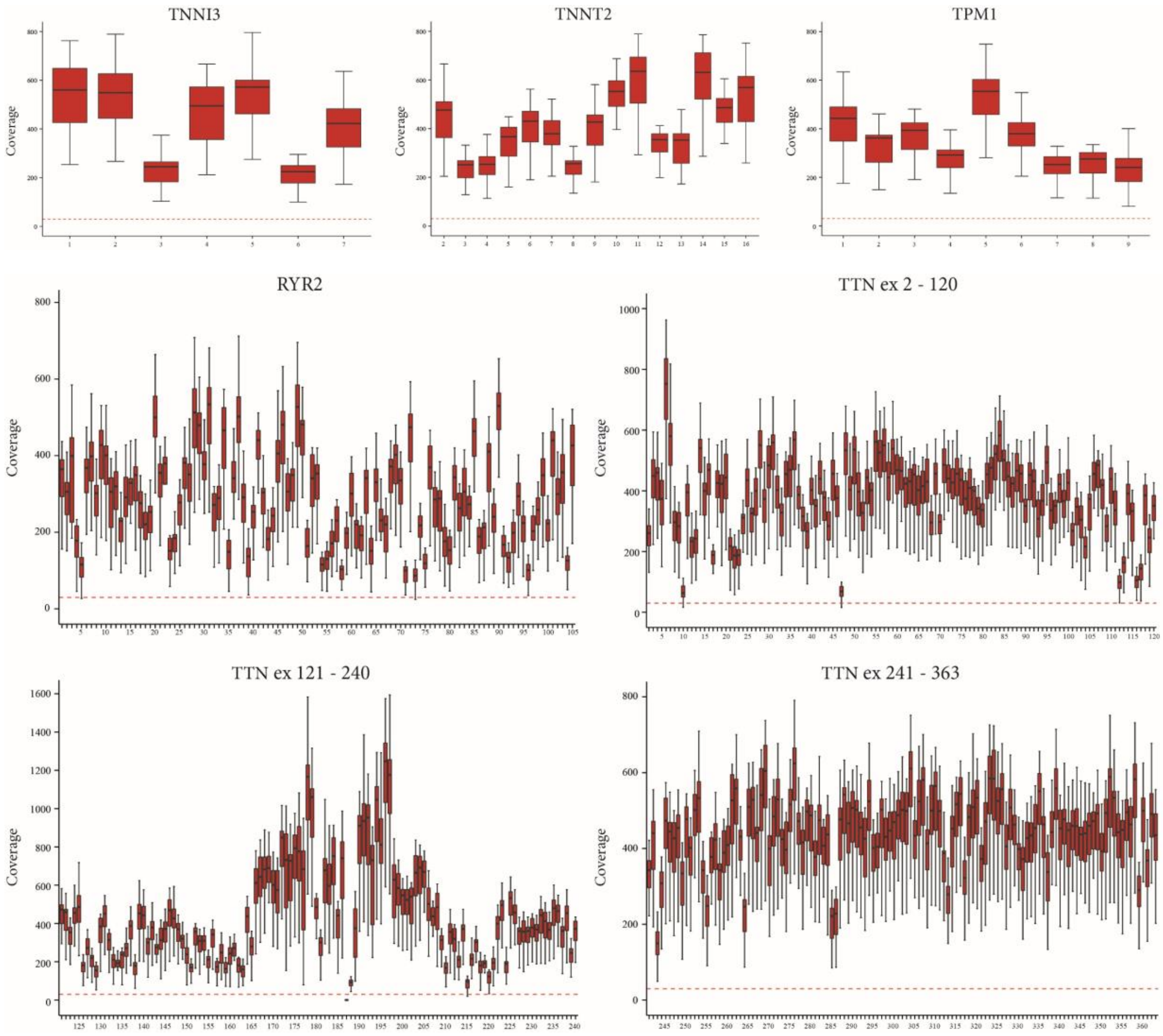

Supplementary Figure S1: Read-depth (coverage) of all exons for all genes from iteration one of the custom-cardiac panel. The dashed red line indicates the 30X read-depth threshold.

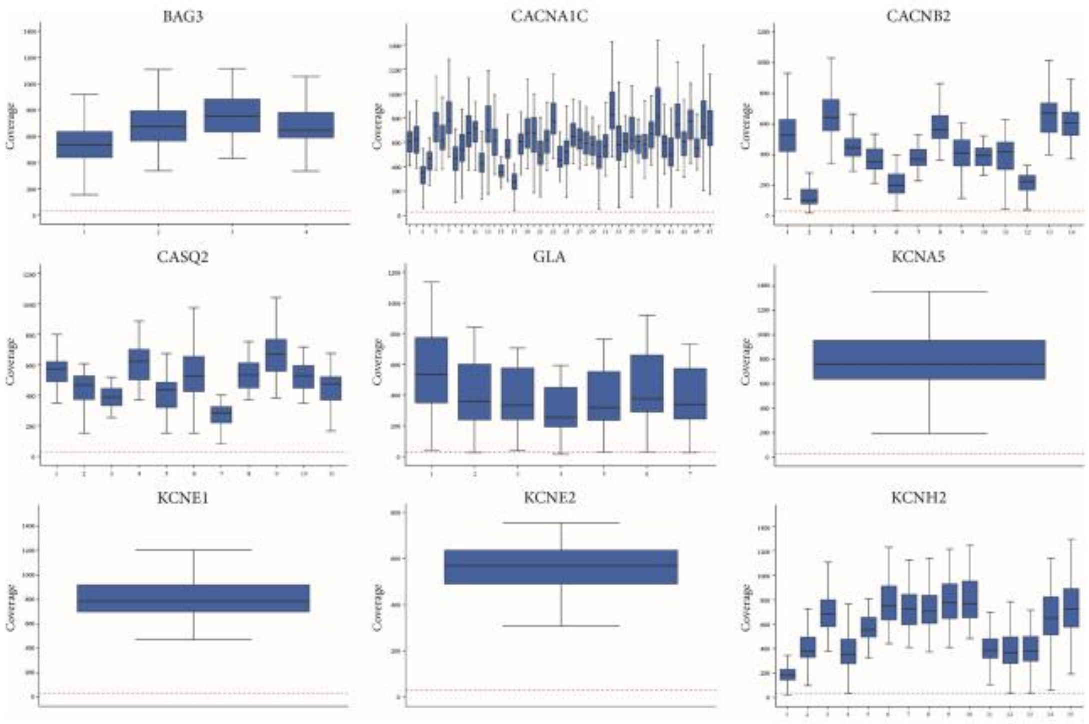
BAG3
CACNA1C
CACNB2
CASQ2
GLA
KCNA5
KCNE1
KCNE2
KCNH2
Coverage

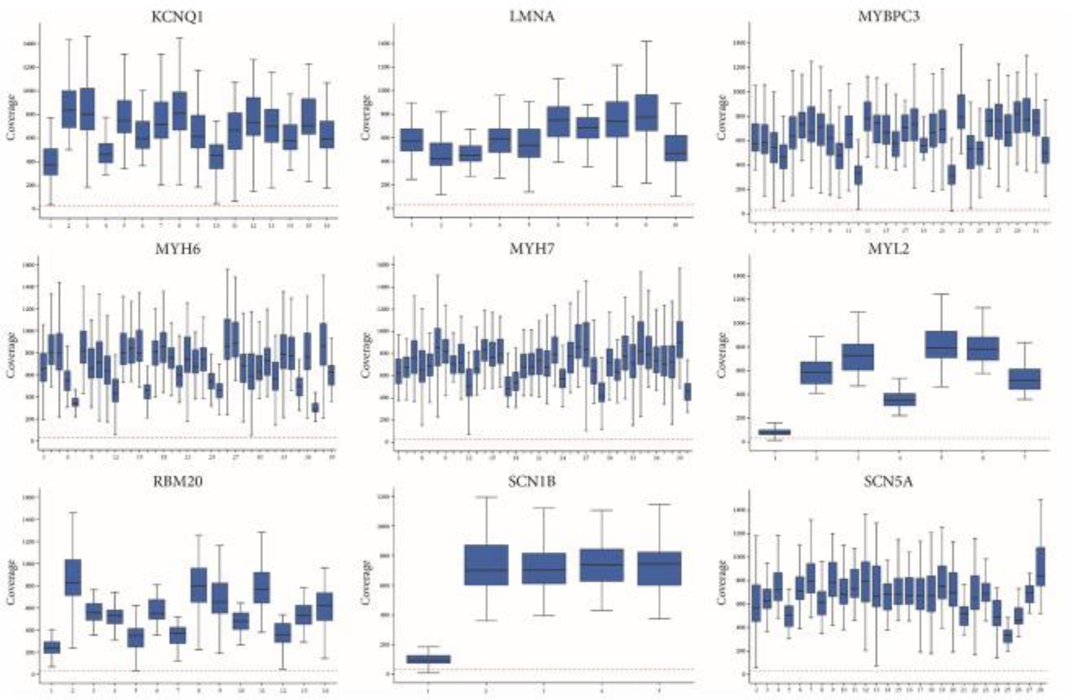
KCNQ1
LMNA
MYBPC3
MYH6
MYH7
MYL2
RBM20
SCN1B
SCN5A
Coverage

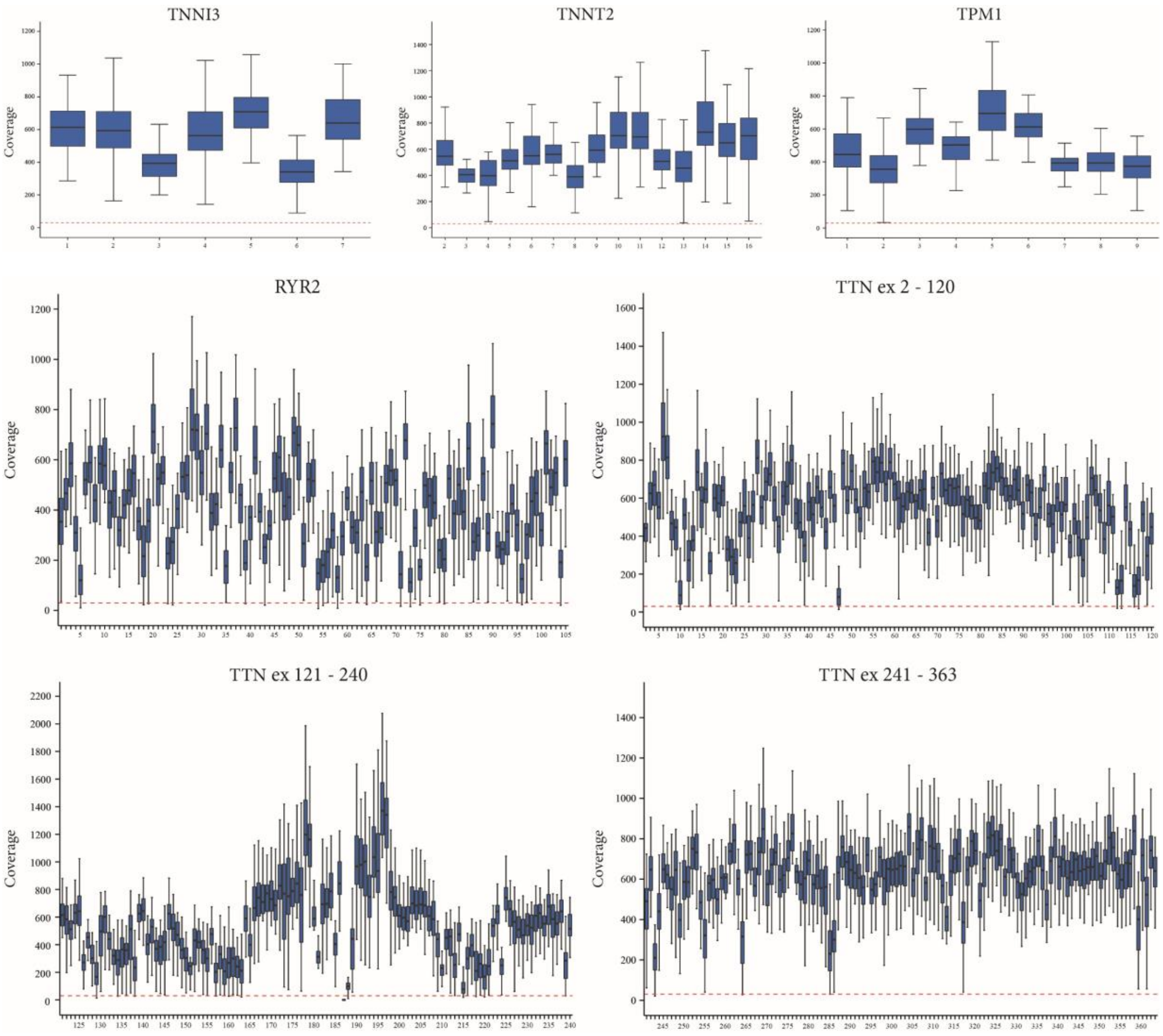

Supplementary Figure S2: Read-depth (coverage) of all exons for all genes from iteration two of the custom-cardiac panel. The dashed red line indicates the 30× read-depth threshold.

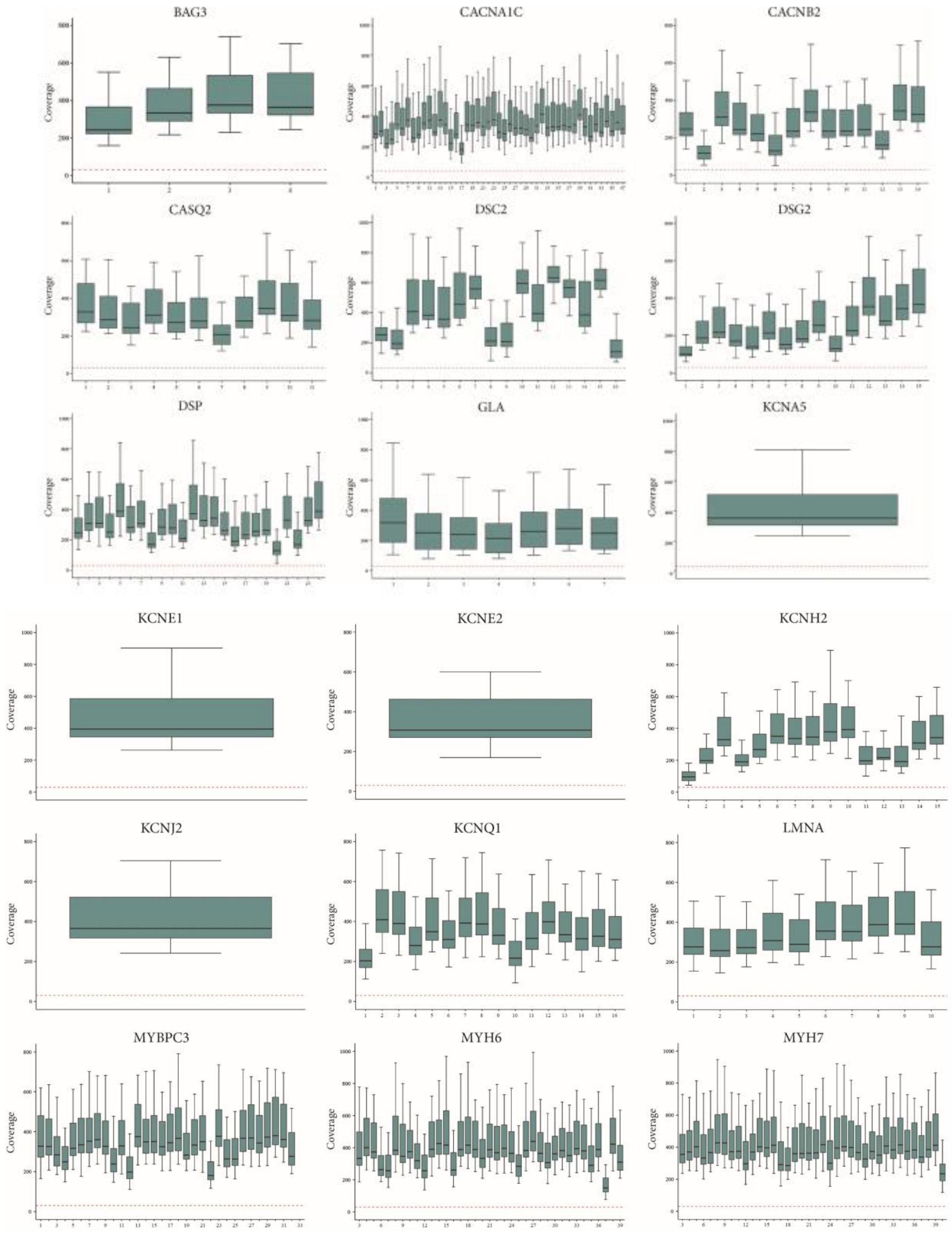
BAG3
CACNA1C
CACNB2
CASQ2
DSC2
DSG2
DSP
GLA
KCNA5
KCNE1
KCNE2
KCNH2
KCNJ2
KCNQ1
LMNA
MYBPC3
MYH6
MYH7
Coverage

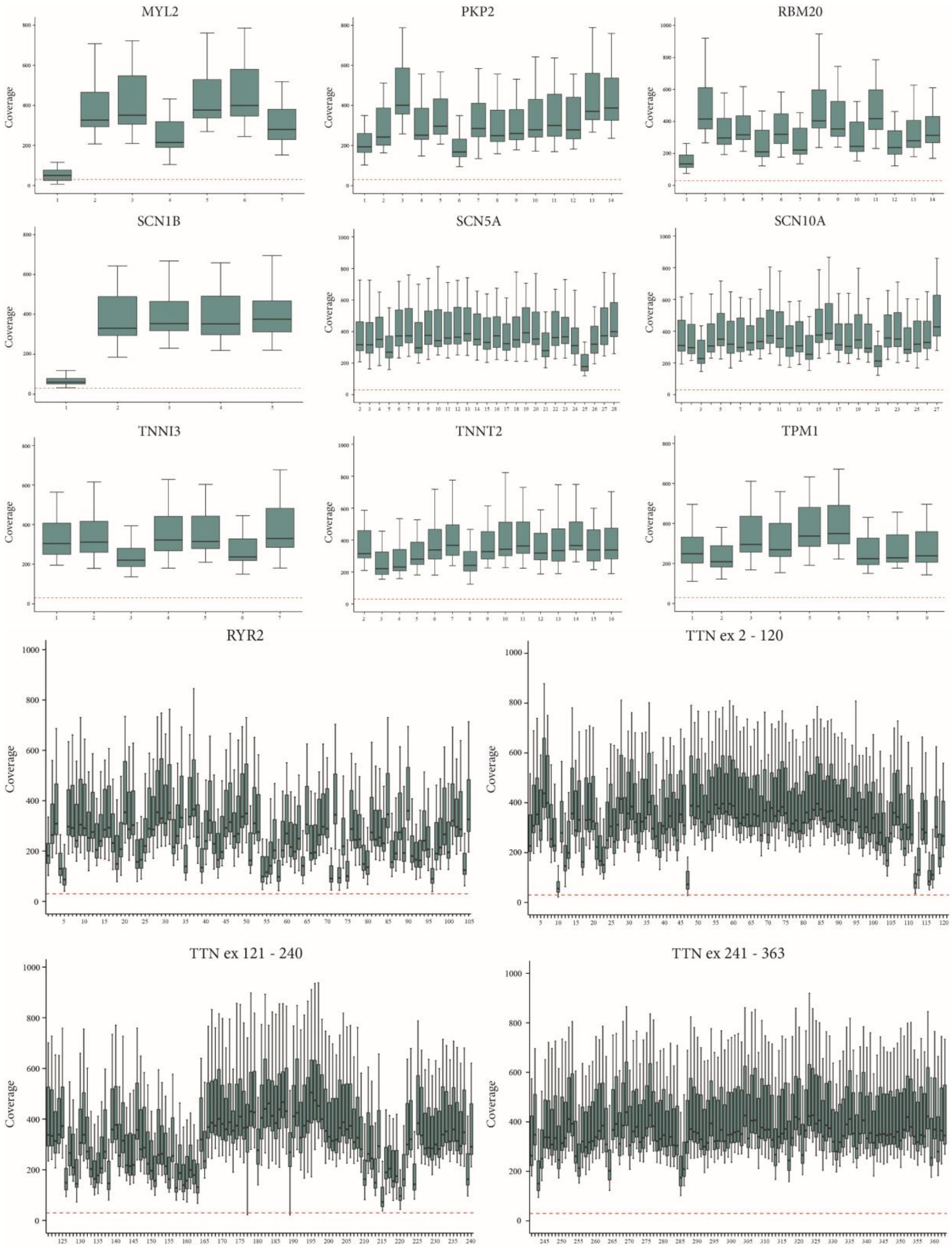

Supplementary Figure S3: Read-depth (coverage) of all exons for all genes from iteration three of the custom-cardiac panel. The dashed red line indicates the 30× read-depth threshold.

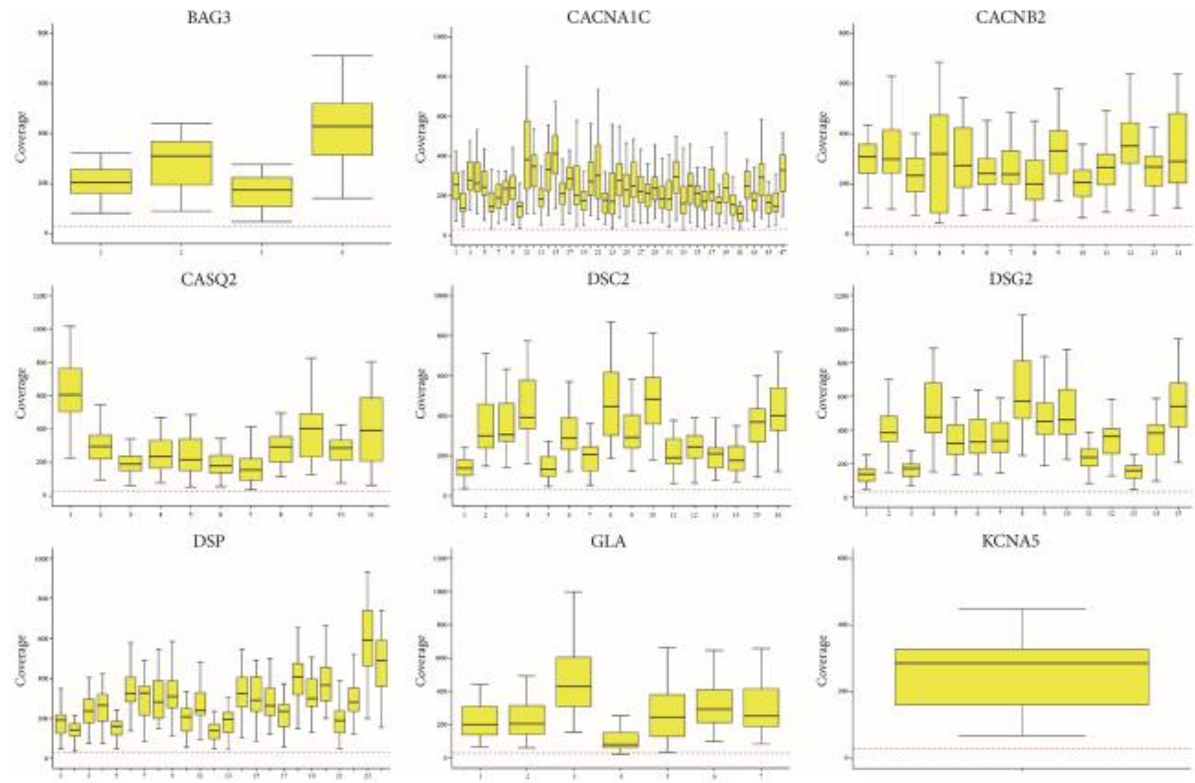
BAG3
CACNA1C
CACNB2
CASQ2
DSC2
DSG2
DSP
GLA
KCNA5
Coverage

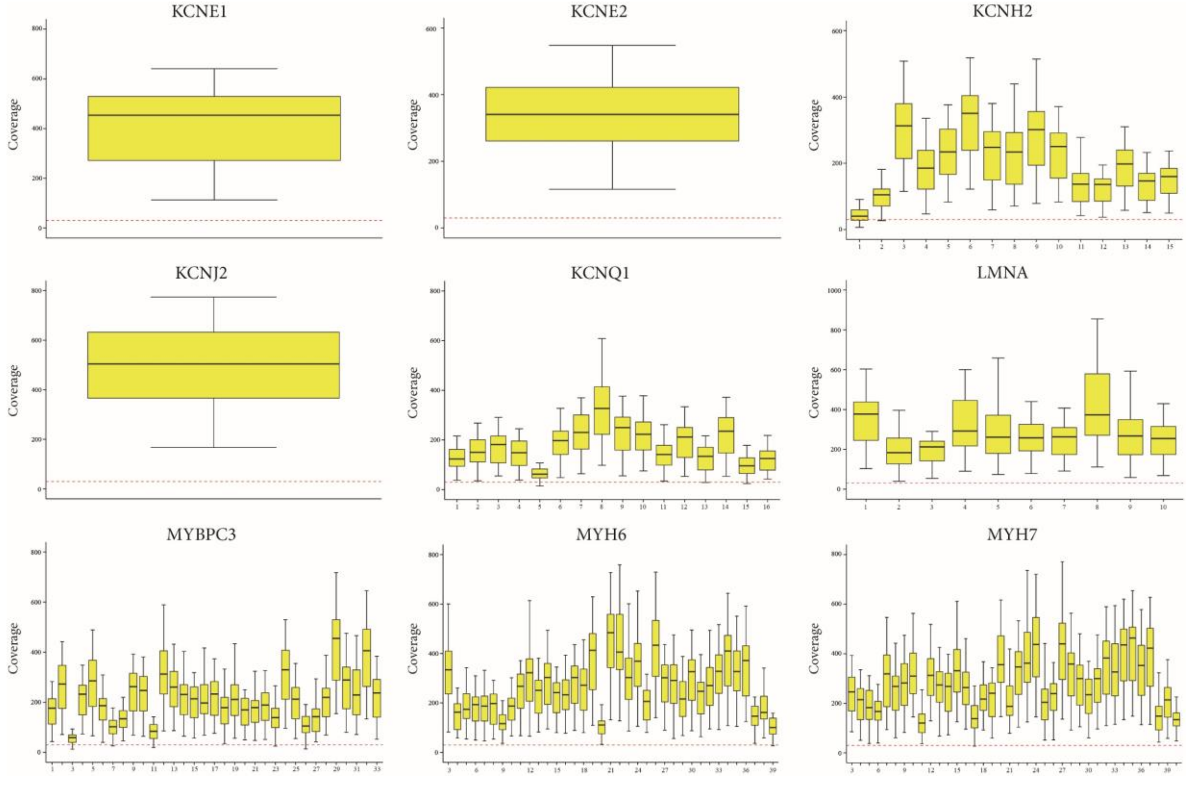
KCNE1
KCNE2
KCNH2
KCNJ2
KCNQ1
LMNA
MYBPC3
MYH6
MYH7
Coverage

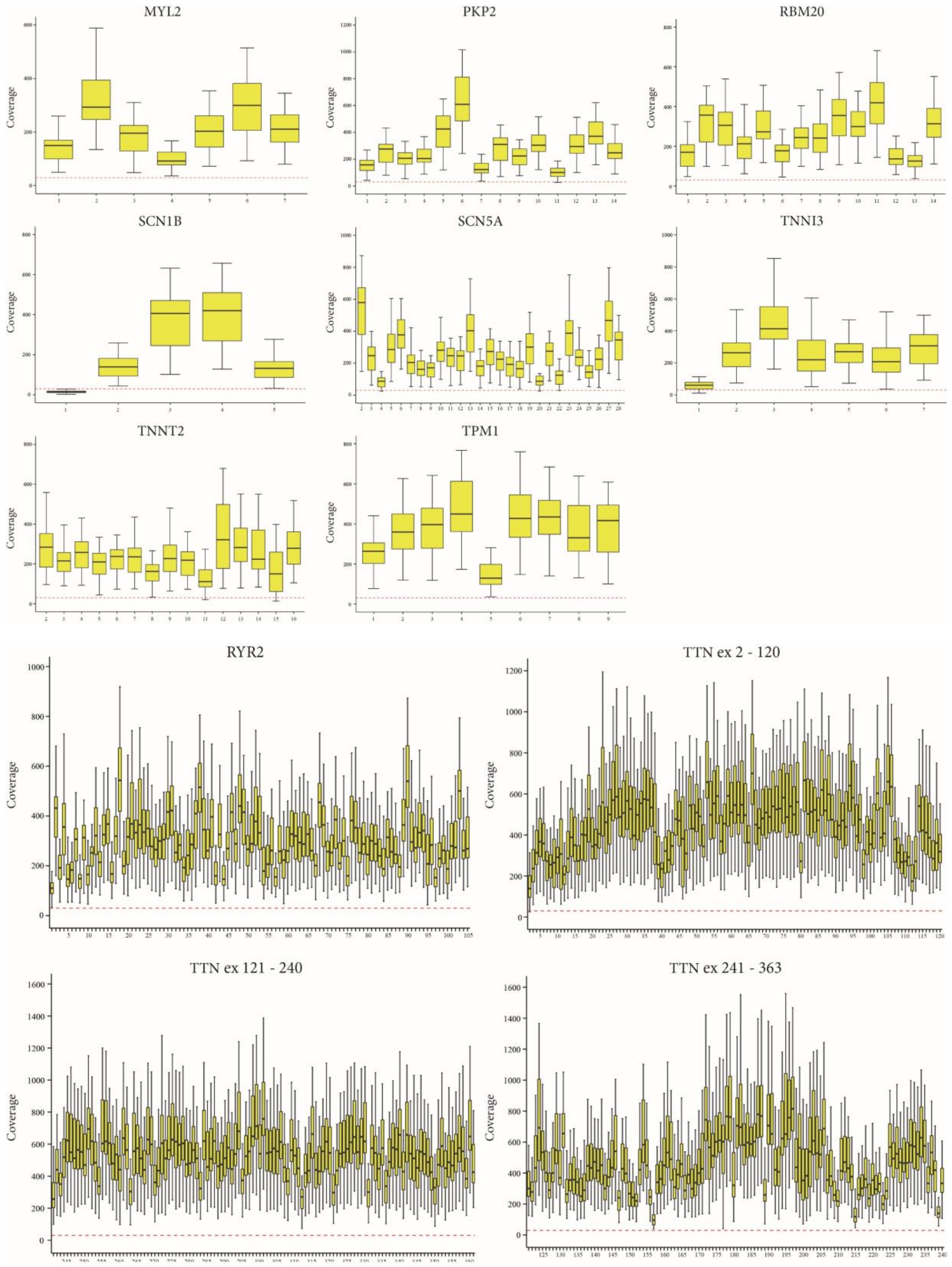

Supplementary Figure S4: Read-depth (coverage) of all exons for all genes that were analysed from the TruSight® Cardio panel. The dashed red line indicates the 30× read-depth threshold.
